# COVID-19 has triggered a new century of vaccination and infection control for the benefit of all mankind

**DOI:** 10.1093/pcmedi/pbab010

**Published:** 2021-06-01

**Authors:** Barry J Marshall

**Affiliations:** Helicobacter pylori Research Laboratory, School of Biomedical Sciences, Marshall Centre for Infectious Disease Research and Training, University of Western Australia, Nedlands 6009, Australia

Modern safe vaccinations were pioneered in 1796 by Edward Jenner in England, when he noticed that milkmaids had beautiful complexions, clear of the blemishes from smallpox scars. This was attributed to their exposure to ‘cowpox' in localised blisters, which seemed to protect them from the more severe and often fatal ‘smallpox'.

In the twentieth century, the importance of immunity was emphasised by the very first Nobel Prize in Medicine, awarded to Emil Adolf von Bering who recognised the therapeutic role of antibodies in blood,^1^ using plasma from a recovered human (or horse) to protect and treat diphtheria, and eventually inventing the diphtheria vaccine in 1907. The first vaccines were simply made, being denatured protein extracts of live cultured bacteria, so there was no danger of causing the disease from the vaccination. Diphtheria-Pertussis-Tetanus (DPT) vaccine has long been available and is given to infants, making these three dreaded diseases of children uncommon in Western countries.

My first personal experience with vaccination was as a 6-year-old (school grade 1) with my mother and 3-year-old brother attending the town hall in Kalgoorlie, Western Australia, for a mass polio vaccination administering the Salk vaccine. I remember that the vaccine was in a 50 ml multiple use bottle containing an estimated 25 dosages of 2 ml. The hall was pandemonium, with lines of people and numerous crying children. Hygiene in the stuffy, packed hall was less than ideal, the multi-use needles simply being soaked in alcohol for sterilisation between patients, becoming blunt and unsafe for use. But there had been at least a 12-month delay before the Salk vaccine could be used in Australia, as one of the early batches from Cutter Labs USA was withdrawn. The virus antigen made from cultured polio virus had not been sterilised adequately in 1955, resulting in more than 250 cases of actual polio in the USA. This caused the FDA to go on high alert, insisting on more stringent manufacturing and quality control procedures, followed by large-scale phase 1, 2 and 3 testing for all new vaccines. The concept is that, because vaccines are given to healthy people, a one-in-a-million incidence of severe side effects (or death) may be too much, even when preventing a dangerous disease such as polio or more recently COVID-19.

Attenuated live polio vaccine replaced the Salk injected vaccine after 1960. Under the umbrella of the school vaccination programme, I received the new format whereby a drop of the pink vaccine was placed on a sugar cube and then eaten. The success of the new Sabin vaccine was its simplicity and oral format. After all, polio is an enterovirus, and I suppose family members could be infected with the live vaccine strain if schoolchildren experienced a very mild gastrointestinal illness at home. The live vaccination trivalent Sabin strain could cause overt polio in very few cases so that, as the actual wild-strain polio became extremely rare, vaccination-strain polio became relatively more common. For that reason, most polio vaccinations are once again using an updated Sabin bivalent vaccine model, reducing the cases of vaccine-caused polio to near zero.^2^

In 1995 I was invited to Philadelphia by Dr Maurice Hilleman, who had developed many of the common vaccines in use today, most notably the Measles Mumps Rubella (MMR) vaccine. He used the unconventional source of his infected daughter to isolate the mumps virus in order to develop the vaccine. That visit opened my eyes to the many possibilities for producing vaccines, from chimeric attenuated virus to nasal inhalations and even the ‘holy grail' of vaccines, that is in food such as transgenic bananas.

Long before receiving a Nobel Prize in 2005 (for *Helicobacter* and Peptic Ulcers), I was awarded the Prince Mahidol medical prize in Thailand. This is the Asia ‘Nobel', which I shared with Prof. Lam Sai Kit for the discovery of Nipah virus spread by bats in Malaysia.^3^ That virus kills about 50% of infected humans by causing encephalitis and pneumonia. Prof. Lam's studies showed that as a result of a weak monsoon, smoky fires on farms in Indonesia had forced a mass unexpected migration of the bats who then colonized fruit trees on pig farms in Malaysia. Fortunately for us, the disease was not easily spread from human pig farmers to other humans and was controlled by destroying all the infected pigs and eliminating contact between the new pigs and the fruit bats. So, that epidemic could be controlled with quarantine (no transporting) and isolation between fruit bats and pigs. Little did we know that the same principles would be needed for the next two human pandemics.

My own observations of the 2003 SARS epidemic were that normal hygiene measures were inadequate for aerosols of infected viral fluids. Face masks and complete face barriers were essential to protect the health professionals. Not only this, but it was also vital that the population understood that hand hygiene was extremely important. After that, and especially during the 2009 swine flu pandemic, hand hygiene facilities became commonplace. However, no-one had taken the time to calculate how many people would need to use these strategies every day for several months, thus the required masks, gloves, and face shields fell into short supply. The fancy technology that was necessary to treat the serious and unstable cases of pneumonia was also not immediately available.

**Figure 1.**
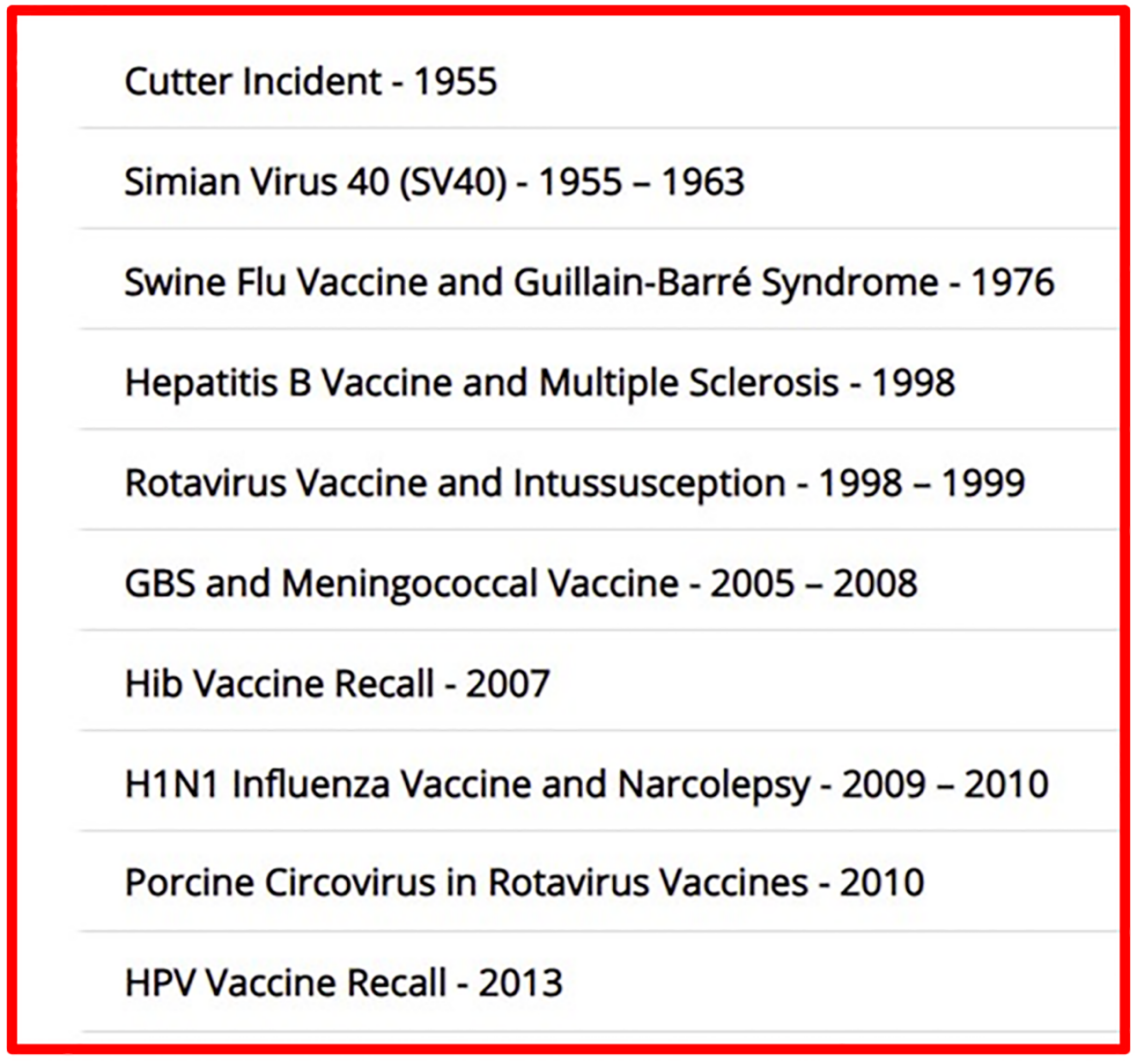
Early vaccine safety withdrawals in USA.^4^

These observations and a quick literature search reminded me that many of the very important vaccines in common use today, had a ‘false start'. No-one need be ashamed if their vaccine technology is a failure. The CDC has reported on the timeline of early safety vaccine withdrawals in the USA (Fig. 1).^4^

This list emphasized to me the importance of the following key ideas:

1. Safety testing in a few hundred animals and humans is probably not enough.
2. Try not to be too hasty with the rollout.
3. Blinded placebo-controlled studies are best.
4. Prepare for a very expensive development cycle with at least one false start.
5. Safety is all important, severe side effects should be less than 0.001% (1/100 000).

In general, a very antigenic vaccine might give excellent protection but might produce a more extreme and long-lasting inflammatory reaction. To some extent, vaccine manufacturers must tread a fine line between efficacy and safety. If the vaccine has few side effects, it may be weaker, with less protection after a few months, and thus will require more booster shots. If no other optimised solutions arise, this becomes the best option. But certainly, a single-dose strong vaccine with no side effects would be preferred. Cost is another issue, as vaccine cost is related to maintenance of the cold chain and the human resources required to administer all those injections. I can see why Hilleman's group was interested in bananas.

The COVID-19 pandemic is a wake-up call for the world's governments. I recall that the cost of the 2003 SARS epidemic was estimated to have been about USD $40B,^5^ mostly to China's economy. The COVID pandemic has cost far more as it has affected every country. We realise now that until everyone is vaccinated and protected, international travel will be curtailed. The Australian government has spent at least AUD $100B (about USD $77.4B) on related costs and we are just 2% of the world's GDP so the global cost must be more than USD $1trillion. Every country needs to rate vaccination technologies on a par with other important parts of infrastructure because we all expect to be free of infectious diseases and to have a predictable but interesting long life. This is impossible if persons from disease-endemic areas cannot travel.

There is reason for optimism now that the COVID-19 pandemic has been well studied and vaccines are becoming available. Firstly, it has been extremely useful that the viral sequence was published from the Wuhan group within a few weeks of the epidemic disease being noted. Because of this, the actual numbers in the pandemic could be accurately tallied, progress could be measured, and vaccine companies could plan how to measure the safety and efficacy of their products.

Secondly, new technologies have been funded by governments, and regulators have sped up the approval progress with emergency measures and increased human resources.^6^ We now have peptide antigens, produced in many ways, most amazingly by the mRNA-type vaccines. This technology promises to give very short lead times for future vaccines. This means the statement that ‘the virus is always changing so a vaccine is not possible' is no longer true. The newer vaccine technologies allow us to track the virus as its genome evolves.

The acceleration of development in innovative technologies under the duress of a global pandemic is already under way and allied with the confidence in our traditional methods of infection control, there is hope for our ability to adapt and overcome future outbreaks. With contact tracing and smartphone applications, whole populations can be alerted to change their behaviour, with daily or even hourly updates. Certainly, education and twenty-first century information flow^7^ are some of our best defences against the next pandemic.
